# Treatment patterns and costs among hospital births with abnormal postpartum uterine bleeding and postpartum hemorrhage in the United States (2016–2022)

**DOI:** 10.1002/pmf2.70050

**Published:** 2025-06-18

**Authors:** Kara M. Rood, Candice Yong, Patricia I. Carney, Brian Seal, Seungyoung Hwang, Krishna Tangirala, Roy M. Brooks

**Affiliations:** ^1^ The Ohio State University‐Wexner Medical Center Columbus Ohio USA; ^2^ Organon & Co. Jersey City New Jersey USA; ^3^ Capital Women's Care Laurel, MD & Holy Cross Hospital Silver Spring Maryland USA

**Keywords:** cesarean section, direct service costs, methylergonovine, morbidity, postpartum hemorrhage, tranexamic acid, uterine balloon tamponade, utilization review

## Abstract

**Background:**

Postpartum hemorrhage (PPH) is the leading cause of maternal morbidity and mortality globally, and PPH rates in the United States are increasing. The current study describes treatment patterns and healthcare costs in abnormal postpartum uterine bleeding and PPH in the United States.

**Method:**

Utilizing data from the US Premier Hospital Database from January 2016 to March 2022, this study investigated treatment patterns for second‐line uterotonics and tranexamic acid (TXA), as well as the incidence of PPH and associated severe maternal morbidity (SMM) events, treatment patterns, and total healthcare costs. The analysis included 5,345,753 inpatient admissions for deliveries, identified PPH through ICD‐10 CM codes or administration of specified uterotonic medications beyond oxytocin.

**Results:**

A total of 787,964 (14.7%) individuals had PPH and/or received second‐line uterotonics beyond oxytocin (methylergonovine maleate, carboprost, misoprostol) and/or TXA. Rates of second‐line uterotonics/TXA and/or PPH increased from 12.1% in 2016 to 19.7% in 2022. Methylergonovine (51.9%) and misoprostol (47.0%) were the most used uterotonics. The mean ± SD SMM and nontransfusion SMM comorbidity scores were 6.8 ± 8.9 and 8.5 ± 12.4, respectively, and included common maternal risk factors such as advanced maternal age, anemia, prior cesarean delivery, and hypertensive disorders. Economic burden was high, particularly for individuals who required hysterectomy (vaginal births: $29,375 and cesarean births: $34,103), received uterine balloon tamponade (UBT; vaginal births: $12,605 and cesarean births: $20,188), and/or were treated with 3 or more second‐line uterotonics/TXA (vaginal births: $10,084 and cesarean births: $16,288). Intensive care unit admissions and hysterectomies were more common in the 3–4 uterotonics/TXA groups (4.4% and 2.2%) and among those who received UBT (9.4% and 4.4%) than those who received < 3 uterotonics/TXA.

**Conclusion:**

Using second‐line uterotonic/TXA use and PPH diagnosis codes, the observed rate of abnormal postpartum bleeding was higher than previously reported and associated with significant clinical and economic burden. Total costs increased with greater use of uterotonics/TXA, especially in cases involving UBT or SMM such as hysterectomy. Higher treatment intensity likely reflects escalation in response to more severe clinical presentations. These findings highlight the need for appropriate intervention, better treatment options, and preventive strategies to manage abnormal postpartum uterine bleeding and PPH.

## 1 INTRODUCTION

Postpartum hemorrhage (PPH) results in 70,000 maternal deaths globally, accounting for 12% of maternal deaths in the United States [1, 2]. Complications of PPH can result in severe maternal morbidity (SMM), which includes serious clinical outcomes such as blood transfusions administered to manage significant blood loss and restore hemodynamic stability, admissions to the intensive care unit (ICU), and hysterectomy. These events are associated with prolonged hospital stays and increased healthcare costs [3, 4].

Although PPH is largely preventable with timely recognition and treatment, the rate of PPH diagnoses in the United States has increased steadily over the past few decades. Previous studies reported a rise from 2.2% in 1994 to 2.9% in 2005 with uterine atony accounting for 79% of PPH cases [5, 6]. More recent data show that the rate further increased from 2.7% in 2000 to 4.3% in 2019 [4]. The economic burden associated with PPH in the United States is ∼1.8 billion dollars, and estimated costs of delivery with complications (including PPH) are higher when compared to those without any complications [7, 8, 9].

Standard management of PPH involves treatment with oxytocin and second‐line uterotonic (UT) medications, fluid resuscitation, and bimanual uterine massage [10, 11]. The use of tranexamic acid (TXA) in PPH management was included in the 2017 WHO guidelines after the publication of the WOMAN study [12, 13]. When these treatments fail, nonsurgical options to address persistent bleeding include uterine packing and uterine balloon tamponade (UBT). In addition, an intrauterine vacuum‐induced hemorrhage‐control device is now available in the United States, though it was not available during the time frame of this analysis [14]. When bleeding persists due to an atonic uterus, a ruptured uterus, or a cervical laceration extending into the uterus, escalation to surgical intervention may be necessary. The overall goal in PPH management is to prevent maternal death while reducing SMM events [13, 15, 16].

Understanding the treatment patterns and economic burden associated with PPH may help to optimize the management of this potentially life‐threatening condition by identifying gaps in clinical practice, guide resource allocation, and support the development of standardized, cost‐effective treatments. While the association of PPH with increased maternal mortality is well established, there are limited recent data on patterns of uterotonic drug utilization in current clinical practice and the impact of PPH on healthcare resource use, including hospital length of stay, transfusion, and hysterectomy. In an analysis of deliveries at select US hospitals from 2007 to 2011, Bateman et al. reported a median hospital‐level frequency of second‐line uterotonic use of 7.1% (interquartile range: 5.2% to 10.8%); however, more recent estimates and data on treatment patterns relative to other interventions are lacking [17].

This study evaluated the treatment patterns, occurrence of SMM events, and associated costs among hospital births involving abnormal postpartum uterine bleeding or PPH in the United States. To further understand the treatment landscape, subgroup analyses for the number of UTs/TXA used, use of UBT, and mode of delivery were conducted.

## 2 METHODS

### 2.1 Study design

This was a retrospective, observational study from the PINC AI™ Healthcare Database (PHD), formerly called the Premier Healthcare Database. The data for deliveries recorded from January 2016 to March 2022 were evaluated. The PHD encompasses a range of hospital‐based services and has information on diagnoses, procedures, and products related to > 326 million unique individuals from > 1190 contributing hospitals and healthcare systems. The PHD represents 25% of all annual inpatient admissions in the United States [18].

Individual demographics including age, race, ethnicity, and payer type were captured in the PHD. Disease states and comorbid conditions are identified using the International Classification of Diseases (ICD) coding system for each encounter. Diagnostic and therapeutic procedures ordered during encounters are identified through ICD codes, Current Procedural Terminology (CPT) codes, and Healthcare Common Procedure Coding System (HCPCS) codes [18].

### 2.2 Study population

The study population consisted of individuals aged 15 years and older with hospital births in the PHD who received at least one second‐line UT beyond oxytocin (methylergonovine maleate, carboprost, misoprostol ≥800 µg) and/or TXA or PPH from January 2016 to March 2022. Second‐line UT use beyond oxytocin, or tranexamic acid use, was a proxy for abnormal postpartum uterine bleeding. Both vaginal and cesarean deliveries, irrespective of term or preterm status, were included in the analyses. Individuals with PPH were identified based on the presence of at least one ICD‐10 code for PPH (O72.0, third‐stage hemorrhage; O72.1, other immediate PPH) during each delivery hospitalization. Those having hospitalizations with codes indicating abortion, ectopic pregnancy, hydatidiform mole, or other abnormal products of conception, as well as deliveries with a gestation period of fewer than 20 weeks, were excluded.

### 2.3 Outcome measures

The analysis of treatment patterns for UTs (methylergonovine maleate, carboprost, misoprostol ≥ 800 µg) and TXA during the index delivery hospitalization was based on billing codes. Nonsurgical treatments (UBT and uterine packing), interventional radiology (uterine artery embolization), and surgical interventions vascular ligation, and hysterectomy were categorized using ICD‐10 codes and/or billing codes. Information about compression sutures was not captured in the Premier database.

Healthcare resource utilization during the index delivery was analyzed, and relevant categories were reported for the full cohort (all individuals, including both individuals with PPH and UTs/TXA use, and those with UTs/TXA use but no PPH diagnosis) and specific subgroups. The healthcare resource utilization measures included total hospital length of stay (LOS), admission to ICU, ICU length of stay, and receipt of blood transfusion.

Total costs were assessed from the hospital perspective based on individual hospital accounting systems. The PHD utilizes each hospital's cost accounting system or Medicare Cost‐to‐Charge Ratios (MCCR) to derive procedural and total costs. For hospitals classified as using a ratio of costs to charges (RCC) approach, Premier teams apply MCCR to the hospital‐reported charge data. These values are then reviewed and validated against internal hospital records to ensure accuracy within accepted variance thresholds. The PHD charge master (CDM) captures itemized hospital services, including medical procedures, equipment, supplies, drugs, and diagnostics, with associated unit costs and quantities billed [18].

Total hospital costs included hospital services, medical procedures, equipment fees, supplies, drugs, and diagnostic evaluations such as imaging and laboratory tests. Total costs comprised variable and fixed costs. Variable cost included expenses that relate directly to or vary with the activity (volume) of the department, such as supplies and hands‐on patient care. Fixed cost included depreciation, management, maintenance, and overhead.

Costs were inflated to October 2022 US dollars using the medical care component of the Consumer Price Index [19]. All‐cause costs, irrespective of their obstetrics‐related nature, were separately reported for the index hospitalization period. Average per delivery episode costs, along with standard deviation, were reported for the delivery hospitalization.

SMM was defined based on complications arising from PPH, including blood transfusions, ICU admissions, and hysterectomy. These outcomes were identified using relevant ICD‐10 diagnosis and procedure codes.

### 2.4 Statistical analyses

Descriptive analyses for categorical variables included frequencies and percentage distributions, covering individual demographics, clinical characteristics, and treatment modalities. Continuous variables were reported with mean, standard deviation, encompassing maternal age, healthcare resource utilization, and costs. Healthcare resource utilization and cost estimates were presented on a population basis for the full cohort.

### 2.5 Ethical consideration

This study reports de‐identified data from electric healthcare data; therefore, the study was exempt from institutional review board approval because it does not constitute research on human subjects (as defined by 45 CFR 46.102).

## 3 RESULTS

### 3.1 Demographics

A total of 5,345,753 unique inpatient admissions with delivery were recorded in the PHD from January 2016 to March 2022 (Figure S1). Among the 787,964 deliveries with a PPH diagnosis and/or use of UTs (beyond oxytocin)/TXA, mean ± SD age was 29.0 ± 6.0 years and 50.7% were non‐Hispanic White. Regarding insurance status, 14.8% had traditional Medicaid, 30.2% had Medicaid managed care, and 37.5% had managed care. Mode of delivery was 65.7% vaginal and 34.1% cesarean, and oxytocin was used in 95.0% of cases (Table 1). In the full cohort of 787,964 individuals, the mean (SD) SMM comorbidity score was 6.8 (± 8.9), and the nontransfusion SMM comorbidity score was 8.5 (± 12.4), indicating a moderate overall burden of maternal health risk factors. In the full cohort, common maternal comorbidities included age over 35 years (18.8%), preexisting anemia (17.3%), and previous cesarean delivery (14.8%). Other notable conditions were preterm birth (12.7%), hypertensive disorders such as preeclampsia without severe features (11.4%), and gestational diabetes (9.3%) (Table 2).

**TABLE 1:** Individual demographic and baseline characteristics.

Parameters	Cohort with PPH and/or UT/TXA (*N* = 787,964)
Mean maternal age ± SD, years	29.0 ± 6.0
Race/Ethnicity, *n* (%)	
Hispanic	150,763 (19.1)
Non‐Hispanic Asian	41,047 (5.2)
Non‐Hispanic Black	113,891 (14.5)
Non‐Hispanic White	399,445 (50.7)
Patient insurance type, *n* (%)	
Medicaid—traditional	116,343 (14.8)
Medicaid—managed care	238,068 (30.2)
Managed care	295,320 (37.5)
Commercial‐Indemnity	87,660 (11.1)
Self‐pay	15,562 (2.0)
Medicare—traditional	2,665 (0.3)
Medicare—managed care	1,939 (0.2)
Other^a^	30,407 (3.9)
Delivery method, n (%)	
Cesarean	268,997 (34.1)
Vaginal	518,050 (65.7)
Unknown	917 (0.1)
Oxytocin use, *n* (%)	748,604 (95.0)

Abbreviations: PPH, postpartum hemorrhage; SD, standard deviation; TXA, tranexamic acid; UT, uterotonic.

^a^ Other includes charity, indigent, workers compensation, direct employer contract, other government payors, and other.

**TABLE 2:** Obstetric comorbidities.

	Full cohort *N* = 787,964	PPH and UT *n* = 136,675	PPH and No UT *n* = 44,550	No PPH and UT *n* = 606,739
Obstetric Comorbidity Score, Mean ± SD				
SMM Comorbidity Score	6.8 ± 8.9	8.1 ± 10.0	8.6 ± 10.6	6.3 ± 8.5
Nontransfusion SMM Comorbidity Score	8.5 ± 12.4	10.2 ± 13.8	10.8 ± 14.9	7.9 ± 11.8
Individual Comorbidity, *n* (%)				
Maternal age ≥ 35 years	147,907 (18.8)	25,511 (18.7)	9626 (21.6)	112,770 (18.6)
Anemia, preexisting	136,655 (17.3)	29,911 (21.9)	9760 (21.9)	96,984 (16.0)
Previous cesarean delivery	116,883 (14.8)	18,010 (13.2)	7979 (17.9)	90,894 (15.0)
Preterm birth (< 37 week gestation)	100,343 (12.7)	16,561 (12.1)	6066 (13.6)	77,716 (12.8)
Preeclampsia without severe features or gestational hypertension	89,762 (11.4)	19,516 (14.3)	6097 (13.7)	64,149 (10.6)
Gestational diabetes mellitus	73,649 (9.3)	12,970 (9.5)	4297 (9.6)	56,382 (9.3)
Major mental health disorder	68,799 (8.7)	13,644 (10.0)	4472 (10.0)	50,683 (8.4)
Preeclampsia with severe features	51,640 (6.6)	11,938 (8.7)	3305 (7.4)	36,397 (6.0)
Asthma, acute or moderate to severe	49,639 (6.3)	10,414 (7.6)	3578 (8.0)	35,647 (5.9)
Substance use disorder	48,005 (6.1)	8731 (6.4)	2875 (6.5)	36,399 (6.0)
Delivery BMI *≥* 40 kg/m^2^	41,551 (5.3)	7419 (5.4)	2995 (6.7)	31,137 (5.1)
Twin or multiple pregnancy	27,494 (3.5)	6060 (4.4)	1573 (3.5)	19,861 (3.3)
Bleeding disorder, preexisting	26,153 (3.3)	5883 (4.3)	1740 (3.9)	18,530 (3.1)
Chronic hypertension	19,251 (2.4)	3983 (2.9)	1373 (3.1)	13,895 (2.3)
Uterine fibroids	16,883 (2.1)	3021 (2.2)	1599 (3.6)	12,263 (2.0)
Preexisting diabetes mellitus	14,610 (1.9)	2463 (1.8)	981 (2.2)	11,166 (1.8)
Placental abruption	14,519 (1.8)	2786 (2.0)	1221 (2.7)	10,512 (1.7)
Gastrointestinal disease	14,158 (1.8)	2788 (2.0)	772 (1.7)	10,598 (1.7)
Cardiac disease, preexisting	11,568 (1.5)	2546 (1.9)	944 (2.1)	8078 (1.3)
Placenta previa, complete or partial	7866 (1.0)	1762 (1.3)	777 (1.7)	5327 (0.9)
Placenta accreta spectrum	3943 (0.5)	1664 (1.2)	981 (2.2)	1298 (0.2)
Neuromuscular disease	4146 (0.5)	806 (0.6)	258 (0.6)	3082 (0.5)
Chronic renal disease	3110 (0.4)	692 (0.5)	224 (0.5)	2194 (0.4)
Connective tissue or autoimmune disease	2125 (0.3)	447 (0.3)	137 (0.3)	1541 (0.3)
Thyrotoxicosis	2306 (0.3)	439 (0.3)	128 (0.3)	1739 (0.3)
HIV or AIDS	670 (0.1)	105 (0.1)	57 (0.1)	508 (0.1)
Pulmonary hypertension	369 (0.0)	82 (0.1)	29 (0.1)	258 (0.0)

Abbreviations: AIDS, acquired immunodeficiency syndrome; BMI, body mass index; HIV, human immunodeficiency virus; PPH, postpartum hemorrhage; SD, standard deviation; SMM, severe maternal morbidity; TXA, tranexamic acid; UT, uterine tamponade.

### 3.2 Rates of abnormal postpartum uterine bleeding or PPH

**FIGURE 1:**
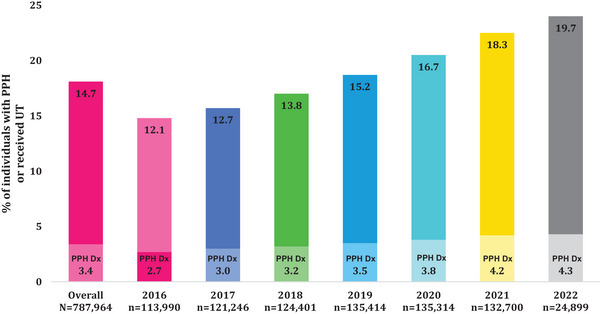
Rates of births with a PPH ICD‐10 diagnosis and/or use of second‐line UTs/TXA (beyond oxytocin) over time. ICD, International Classification of Diseases; PPH, postpartum hemorrhage; PPH Dx, proportion of individuals with ICD‐10 diagnosis code for PPH (± UTs/TXA); TXA, tranexamic acid; UT, uterotonic.

The observed rate of second‐line UTs/TXA use and/or PPH diagnosis was 14.7% (Figure 1); 11.3% of the population received UTs/TXA without a PPH diagnosis code, 2.6% had both a PPH diagnosis code and UTs/TXA administration, and 0.8% had a PPH diagnosis only. There was an increased rate of second‐line UTs/TXA and/or PPH over time, from 12.1% in 2016 to 19.7% in 2022. Likewise, the proportion of individuals with an ICD‐10 diagnosis for PPH increased from 2.7% in 2016 to 4.3% in 2022. Moreover, the rates of second‐line UTs/TXA increased from 9.4% in 2016 to 15.4% in 2022.

### 3.3 Treatment patterns

**FIGURE 2:**
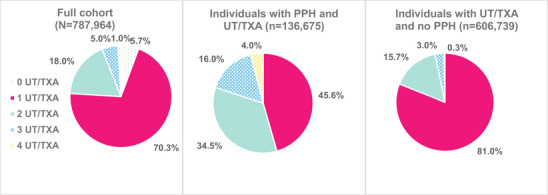
Treatment patterns with UTs/TXA combinations. PPH, postpartum hemorrhage; TXA, tranexamic acid; UT, uterotonic.

For 70.3% of the population, a single UT (beyond oxytocin) and/or TXA was the most common treatment approach; however, individuals with a PPH diagnosis were more likely to receive multiple UTs/TXA combinations (Figure 2). When multiple UTs/TXA were used, 18% of individuals received two combinations and 6.0% received combinations of three or more. Methylergonovine maleate was the most commonly used second‐line UT (51.9%), followed by misoprostol ≥800 µg (47%) and carboprost (15.4%); TXA was used in 11.1% of cases (Table 3). Among nonsurgical interventions, UBT and uterine packing were used in 2.7% and 2.1% of individuals, respectively. UBT use was more frequent in the PPH and UT cohort compared with the no PPH and UT cohort (11.6% vs. 0.6%). Surgical intervention with hysterectomy or uterine artery embolization was performed in 0.6% and 0.3% of individuals, respectively. Individuals in the PPH and UT cohort had a higher rate of hysterectomy (1.8%) compared with the no PPH and UT cohort (0.2%). Among individuals who received both UBT and pharmacologic therapy, combination therapy with 3 or more UTs/TXA increased from 26.6% in 2016 to 52.4% in 2022 (Figure S2).

**TABLE 3:** Treatment patterns with uterotonics and/or tranexamic acid and other interventions.

Treatment, *n* (%)	Full Cohort	PPH and UT/TXA	No PPH and UT/TXA
	*N* = 787,964	*N* = 136,675	*N* = 606,739
UTs/TXA			
Methylergonovine maleate	409,268 (51.9)	85,439 (62.5)	323,829 (53.4)
Carboprost	121,119 (15.4)	44,771 (32.8)	76,348 (12.6)
Misoprostol (total dose ≥800 µg)	370,042 (47.0)	81,893 (59.9)	288,149 (47.5)
TXA	87,160 (11.1)	31,767 (23.2)	55,393 (9.1)
Nonsurgical treatment			
Uterine balloon tamponade	20,948 (2.7)	15,911 (11.6)	3536 (0.6)
Uterine packing	16,546 (2.1)	3511 (2.6)	12,203 (2.0)
Surgical/radiologic intervention			
Uterine artery embolization	2296 (0.3)	1428 (1.0)	538 (0.1)
Hysterectomy	4650 (0.6)	2490 (1.8)	1068 (0.2)
Vascular ligation	3 (0.0)	2 (0.0)	1 (0.0)

Abbreviations: PPH, postpartum hemorrhage; TXA, tranexamic acid; UT, uterotonic.

### 3.4 Economic burden

**FIGURE 3:**
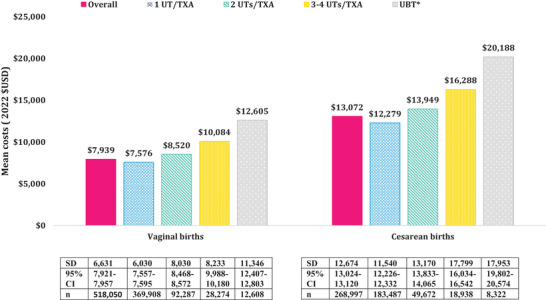
Total costs during index hospitalization for individuals who received UTs/TXA and UBT. *UBT is a subgroup of the full cohort with/without UTs/TXA. SD, standard deviation; TXA, tranexamic acid; UBT, uterine balloon tamponade; USD, United States dollar; UT, uterotonic.

Overall, the mean total cost during the index hospitalization was $9693 ($15,617 with UBT and $9531 without UBT) (Table S3). By birth mode, costs were $7939 for vaginal and $13,072 for cesarean births. In both vaginal and cesarean births, mean total costs were highest for individuals who received UBT, followed by those who received 3 to 4 UTs/TXA (Figure 3). Mean total costs during index hospitalization for individuals who received UBT were $12,605 in vaginal births and $20,188 in cesarean births. In the cesarean cohort, the total costs increased with the number of UT/TXA administrations, ranging from $12,279 (1 UT/TXA) to $16,288 (3‐4 UTs/TXA). Similarly for vaginal deliveries, the mean total costs ranged from $7576 with 1 UT/TXA to $10,084 with at least 3 UTs/TXA administered. The primary cost driver for both vaginal and cesarean deliveries was room and board in the ICU ($5241 and $7075, respectively), followed by surgery ($2214 and $3636), and labor and delivery expenses ($1583 and $1755) (Table S2).

### 3.5 Hospital LOS

In the full cohort regardless of UBT status or UT/TXA use, the mean ± SD LOS was 2.4 ± 1.8 days for vaginal births and 2.9 ± 2.7 days for cesarean births (Table S1). The UBT cohort had a longer LOS compared to the no‐UBT cohort for both vaginal (3.0 ± 2.3 vs. 2.4 ± 1.8 days) and cesarean (4.6 ± 4.5 vs. 3.8 ± 3.6 days) births. In individuals without UBT, LOS was approximately 1 day longer for cesarean versus vaginal births. Similarly, in individuals with UBT, a higher LOS was observed for cesarean versus vaginal births. Use of UTs/TXA did not have a substantial impact on LOS; however, LOS tended to be longer in the 3‐4 UT/TXA groups.

### 3.6 Severe maternal morbidity outcomes and associated costs

**FIGURE 4:**
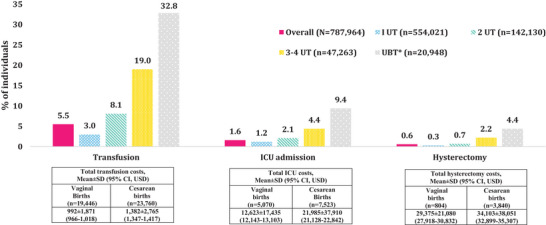
Severe maternal morbidity events and associated costs. *UBT is a subgroup of the full cohort with/without UTs/TXA. ICU, intensive care unit; SD, standard deviation; TXA, tranexamic acid; UBT, uterine balloon tamponade; USD, United States dollar; UT, uterotonic.

Overall, 5.5% of individuals received transfusions, 1.6% were admitted to the ICU, and 0.6% underwent hysterectomy; however, SMM events varied by the number of UTs/TXA received (Figure 4). Blood transfusion ranged from 3.0% in 1 UT/TXA cohort to 19.0% in 3–4 UTs/TXA cohort, admission to the ICU ranged from 1.2% in 1 UT/TXA cohort to 4.4% in 3–4 UTs/TXA cohort, and hysterectomy ranged from 0.3% in 1 UT/TXA cohort to 2.2% in 3–4 UTs/TXA cohort. In the vaginal birth cohort, the highest costs were associated with hysterectomy ($29,375), followed by ICU admissions ($12,623), and transfusions ($992). Similarly, in the cesarean cohort, mean costs were $34,103 for cases with hysterectomy, $21,985 for ICU admissions, and $1382 for transfusions (Figure 4).

## 4 DISCUSSION

The study sample included more than 5.3 million unique inpatient admissions for delivery in the United States. Of these, 14.7% received second‐line UTs/TXA beyond oxytocin and/or had diagnosis of PPH. The most common treatment pattern was the use of a single second‐line UT medication (beyond oxytocin) or TXA; however, individuals with a PPH diagnosis code were more likely to receive multiple UTs/TXA. SMM events were more frequently observed when 3–4 UTs/TXA were administered (ICU admission, 4.4%; hysterectomy, 2.2%) and in those who received UBT (ICU admission, 9.4%; hysterectomy, 4.4%). Economic burden from a hospital perspective was high in the vaginal and cesarean cohorts, particularly total costs for individuals who received UBT (vaginal births: $12,605 and cesarean births: $20,188) and also for those with increased use of multiple UT/TXA combinations (vaginal births: $10,084 and cesarean births: $16,288). In the vaginal and cesarean cohorts, the total costs were highest for cases requiring hysterectomy ($29,375 and $34,103, respectively), followed by ICU admissions ($12,623 and $21,985, respectively), and transfusions ($992 and $1382, respectively).

The data from 787,964 inpatient deliveries included in this nationwide study confirms other reports that the incidence of obstetric hemorrhage is increasing in the United States, despite being largely preventable with timely diagnosis and intervention. We observed an increase from 2.7% in 2016 to 4.3% in 2022 in the proportion of deliveries with an ICD‐10 diagnosis code for PPH. Our findings are consistent with an earlier cross‐sectional study of the National Inpatient Sample database, where rates of PPH defined by ICD codes were 2.7% in 2000 to 4.3% in 2019 [4]. When we expanded the definition of PPH to include use of second‐line UTs/TXA, the obstetric hemorrhage rate increased from 12.1% in 2016 to 19.7% in 2022. Using a similar definition, a recent study from a large academic health system in New York reported a PPH rate of 11% [7]. Treatment patterns evolved during the observation period, with decreasing use of methylergonovine and increasing use of TXA and combination therapy; increased TXA use coincided with the WOMAN trial and guideline update [12, 13]. Together, these findings emphasize the critical need for heightened awareness, early diagnosis, and targeted interventions to address the escalating burden of obstetric hemorrhage and ultimately prevention of SMM.

Our study also augments the limited data on the escalating economic burden of PPH in the United States, particularly when cases involve SMM events such as transfusion or hysterectomy [9]. We observed a pattern of increased total healthcare costs with increasing number of UTs and/or TXA administered for both vaginal and cesarean births, reflecting the cost of polytherapy and likely the severity of cases. The economic impact was further escalated with use of UBT, particularly in cesarean births, where costs reached a peak of $21,159 in UBT recipients. Cesarean births consistently demonstrated higher overall costs compared with vaginal births, notably in ICU‐related expenses. In our study, the total costs associated with hysterectomy ranged from $29,375 in vaginal births to $34,103 in cesarean births. These findings supplement those of a retrospective observational study conducted from January 2019 through March 2020, which reported an average variable direct cost ranging from $8,689 for each PPH case involving any UT administration beyond oxytocin to $29,456 for cases involving 4 or more units of blood or hysterectomy (vaginal and cesarean births combined) [7]. Variable direct costs for cases involving transfusion of 1–3 units of blood were $14,364, with costs of $649 (1–3 units) and $2096 (4 or more units) for blood product [7]. Of note, cost estimates between our study and others may vary due to differences in study designs and the type of costs reported.

Study findings highlight a need for appropriate intervention and improved treatment options to more effectively manage obstetric hemorrhage and ultimately improve individual outcomes. It may be possible to contain the economic burden associated with SMM by preventing or reducing some of these sequelae. In both the vaginal and cesarean cohorts, individuals treated with hysterectomy and UBT incurred the highest costs, reinforcing the economic burden associated with progression to more severe obstetric hemorrhage and longer hospital stays.

These findings prompt further exploration into the efficacy, safety, and cost implications of alternative treatment modalities, as well as the potential for optimizing treatment algorithms to improve individual outcomes and reduce healthcare costs. Future research efforts should focus on identifying cost‐saving measures and optimizing resource utilization to alleviate the financial strain on healthcare systems. Moreover, findings suggest the need to explore and address the under‐recording of PPH diagnosis codes, given that individuals without such codes received multiple drugs intended for managing obstetric bleeding and transfusions.

A strength of this study is that it utilized data from a hospital‐based, nationally representative health network, effectively minimizing selection bias and ensuring robust external validity. This study had a few limitations, as with any other retrospective observational study.

One key limitation is that the database does not capture the clinical indication—whether prophylactic or therapeutic—for the use of second‐line UTs/TXA. The database lacked specific indications (treatment or prophylactic) for the use of second‐line UTs/TXA, leading to an assumption that higher doses of misoprostol (> 800 mcg) were prescribed for cases involving postpartum bleeding. Additionally, procedures such as uterine artery embolization and the use of UBT were identified based on standard hospital discharge billing files and medical procedure codes; this method may result in underestimation of the frequency of these interventions, particularly if not coded consistently or comprehensively. Information about compression sutures was not available in the database and may be used more frequently than either embolization or uterine artery ligation at many institutions. Another limitation inherent to database studies is the reliance on the use and accuracy of medical and procedural codes. While these codes are validated by the data processor for data completeness, consistency, and accuracy, complete outcome and exposure verification using medical records would be impractical due to sample size. This study did not account for potential changes in maternal risk profiles, comorbidities, hospital‐level factors, or billing and coding practices over time, which may have influenced the observed trends in PPH diagnosis and treatment patterns.

## 5 CONCLUSIONS

Using both second‐line UT/TXA use and PPH diagnosis codes as proxies for abnormal postpartum bleeding, we found that the observed rate of abnormal postpartum uterine bleeding was higher than previously reported and associated with substantial clinical and economic burden. Total costs were higher with increasing number of UTs/TXA for both vaginal and cesarean births and when cases involved UBT and SMM events such as hysterectomy. Higher treatment intensity likely reflects escalation in response to more severe clinical presentations. Overall, these results highlight the need for appropriate intervention, improved treatment options, and preventive strategies to better manage obstetric bleeding and reduce the burden of PPH.

## CONFLICT OF INTEREST STATEMENT

Kara M. Rood: RUBY Investigator, Advisory Board participation, paid consultant (Organon), Advisory Board participation (Cooper Surgical); Candice Yong, Patricia I. Carney, Brian Seal, and Seungyoung Hwang: Organon employees and stock/shareholders; Roy M. Brooks: paid consultant (Organon).

## Supporting information



Supporting Information
