# Supplementary material for: Treatment patterns and costs among hospital births with abnormal postpartum uterine bleeding and postpartum hemorrhage in the United States (2016–2022)
Source: Pregnancy (Hoboken). 2025 Jun 18;1(4):e70050. doi: 10.1002/pmf2.70050 (PMC13344630; doi:10.1002/pmf2.70050)
Supplement: Supplementary file 1 [file PMF2-1-e70050-s001.docx]

# Supplementary Materials Supplemental Table 1. Hospital LOS for index delivery by number of UTs/TXA and with or without UBT

| **LOS, Mean ± SD** | **Full Cohort** | **1 UT/TXA** | **2 UTs/TXA** | **3-4 UTs/TXA** |
| --- | --- | --- | --- | --- |
| **All (UBT or no UBT)** |  |  |  |  |
| Full cohort | 2.9 ± 2.7 | 2.8 ± 2.5 | 3.0 ± 2.7 | 3.3 ± 3.1 |
| Vaginal deliveries | 2.4 ± 1.8 | 2.4 ± 1.7 | 2.5 ± 1.8 | 2.7 ± 1.9 |
| Cesarean deliveries | 2.9 ± 2.7 | 2.8 ± 2.5 | 3.0 ± 2.7 | 3.3 ± 3.1 |
| **UBT** |  |  |  |  |
| Full cohort | 3.6 ± 3.5 | 3.6 ± 3.8 | 3.6 ± 3.3 | 3.6 ± 3.4 |
| Vaginal deliveries | 3.0 ± 2.3 | 2.9 ± 2.5 | 3.0 ± 2.3 | 3.0 ± 2.3 |
| Cesarean deliveries | 4.6 ± 4.5 | 4.6 ± 4.9 | 4.6 ± 4.2 | 4.6 ± 4.4 |
| **No UBT** |  |  |  |  |
| Full cohort | 2.9 ± 2.6 | 2.8 ± 2.5 | 3.0 ± 2.7 | 3.2 ± 3.0 |
| Vaginal deliveries | 2.4 ± 1.8 | 2.4 ± 1.7 | 2.5 ± 1.7 | 2.6 ± 1.9 |
| Cesarean deliveries | 3.8 ± 3.6 | 3.7 ± 3.5 | 3.9 ± 3.7 | 4.0 ± 4.0 |

LOS, length of stay; SD, standard deviation; TXA, tranexamic acid; UBT, uterine balloon tamponade; UT, uterotonic

**Supplementary Table 2. Total costs for specific components of care during index hospitalization**

| **Costs during index hospitalization,  Mean ± SD (USD)**  **95%CI** | **Vaginal deliveries** | **Cesarean section deliveries** |
| --- | --- | --- |
| Room and board ICU | N=5,070  5,241 ± 7,404  5037-5,445 | N=7,523  7,075 ± 14,268  6,753-7,397 |
| Room and board non-ICU | N=515,878  3,173 ± 3,155  3,164-3,182 | N=267,886  4,863 ± 5,999  4,840-4,886 |
| Surgery | N=479,300  2,214 ± 2,529  2,207-2,221 | N=266,878  3,636 ± 3,657  3,622-3,650 |
| Labor and delivery | N=262,943  1,583 ± 1,986  1,575-1,591 | N=102,479  1,755 ± 3,226  1,735-1,775 |
| Other | N=454,198  842 ± 1,599  837-847 | N=261,939  1,227 ± 2,905  1,216-1,238 |
| Recovery room | N=165,295  681 ± 3,378  665-697 | N=202,905  891 ± 1,292  885-897 |
| Pharmacy | N=518,017  452 ± 1,126  449-455 | N=268,991  826 ± 2,007  818-834 |
| Central supply | N=273,631  276 ± 1,026  272-280 | N=219,310  645 ± 1,765  638-652 |
| Laboratory | N=514,917  166 ± 572  164-168 | N=268,725  236 ± 529  234-238 |
| Blood bank | N=468,712  174 ± 592  172-176 | N=255,146  318 ± 1,177  313-323 |

ICU, intensive care unit, SD, standard deviation, USD, United States dollar

**Supplementary Table 3. Total Costs during the index hospitalization**

| **Total cost (USD),**  **Mean ± SD**  **95% CI** | **Full cohort** | **1 UT/TXA** | **2 UTs/TXA** | **3-4 UTs/TXA** |
| --- | --- | --- | --- | --- |
| **All** | 9,693 ± 9,498  9,672-9714 | 9,136 ± 8,603  9,113-9,159 | 10,419 ± 10,451  10,365-10,473 | 12,574 ± 13,302  12,454-12694 |
| UBT | 15,617 ± 14,809  15,416-15818 | 14,365 ± 15,672  13,915-14,815 | 15,289 ± 13,890  14,948-15,630 | 16,600 ± 14,807  16,283-16,917 |
| No UBT | 9,531 ± 9,258  9,510-9,552 | 9,091 ± 8,504  9,069-9,113 | 10,189 ± 10,204  10,135-10,243 | 11,705 ± 12,788  11,578-11832 |

SD, standard deviation; TXA, tranexamic acid; UBT, uterine balloon tamponade; USD, United States dollar; UT, uterotonic

**Supplementary Figure 1. Study population**

**Total number of unique inpatient admissions with delivery
(Jan 2016–Mar 2022)**

**N=5,520,565**

**Age ≥ 15 at delivery**

**N=5,510,540**

**Excluded: Deliveries with any codes indicating abortion, ectopic pregnancy, hydatidiform mole, or other abnormal products of conception**

**N=5,500,832**

**Excluded: Deliveries with <20 weeks or unknown gestation period**

**N=5,345,753**

**Number of individuals with PPH diagnosis OR use of second-line UT/TXA beyond oxytocin**

**N=787,964 (14.7%)**

**PPH and UT/TXA**

**2.6% (n=136,675)**

**PPH without UT/TXA**

**0.8% (n=44,550)**

**UT/TXA without PPH**

**11.3% (n=606,739)**

PPH, postpartum hemorrhage; TXA, tranexamic acid; UT, uterotonics

**Supplementary Figure 2. UT/TXA (beyond oxytocin) treatment patterns over time among individuals who received UBT**

% of UT/TXA use among individuals who received UBT

|  | 1 UT/TXA (%) | 2 UT/TXA (%) | ≥3 UTs/TXA (%) |
| --- | --- | --- | --- |
| 2016 n=2394 | 35.3 | 38 | 26.6 |
| 2017 n=2,645 | 29.1 | 40.4 | 30.4 |
| 2018 n=3,035 | 25.1 | 35.3 | 39.6 |
| 2019 n=3,666 | 23.4 | 30.6 | 46.1 |
| 2020 n=3,490 | 18.7 | 28.9 | 52.4 |
| 2021 n=3,623 | 18.4 | 28.7 | 53 |
| 2022 n=594 | 18.7 | 29 | 52.4 |

TXA, tranexamic acid; UBT, uterine balloon tamponade; UT, uterotonics
